# Silicon Nanoparticles (SiNPs) Enhance Elongation and Rooting of In Vitro Shoots of Vanilla (*Vanilla planifolia* Andrews) During Micropropagation in RITA^®^ Bioreactors

**DOI:** 10.3390/plants14243732

**Published:** 2025-12-07

**Authors:** Marco A. Ramírez-Mosqueda

**Affiliations:** Centro Nacional de Recursos Genéticos-INIFAP, Boulevard de la Biodiversidad No. 400 Rancho las Cruces, Tepatitlán de Morelos 47600, Jalisco, Mexico; marcomosqueda02@hotmail.com

**Keywords:** in vitro propagation, silicon, biostimulant, nanomaterials, vanilla

## Abstract

Vanilla (*Vanilla planifolia* Andrews) cultivation is globally relevant due to the extraction of vanillin from its cured fruits. However, the high demand for propagules for commercial plantations requires new propagation methodologies, including in vitro propagation. Currently, the use of biostimulants in plant micropropagation protocols is being explored to increase the number of plants obtained and their vigor. Nanomaterials such as silicon dioxide nanoparticles (SiNPs) have shown a positive effect on plant growth and development. The objective of this study was to evaluate the effect of SiNPs on the micropropagation of *V. planifolia* in RITA^®^ bioreactors. In vitro plants were transferred to Murashige and Skoog (MS) medium supplemented with different concentrations of SiNPs < 50 nm (0, 50, 100, and 150 mg L^−1^) in RITA^®^ bioreactors. The obtained plants were then acclimatized in a greenhouse. The results indicated that 150 mg L^−1^ of SiNPs produced the highest average shoot number, with 5.12 shoots per explant (5.48 cm in length), 9.50 leaves, and 5.00 roots per explant. The formation of an optimal root system in plants with SiNPs allowed for 98% survival. Results will enable more efficient in vitro propagation protocols through the obtainment of plants with greater length and a developed root system that facilitates ex vitro adaptation.

## 1. Introduction

**Figure 1:**
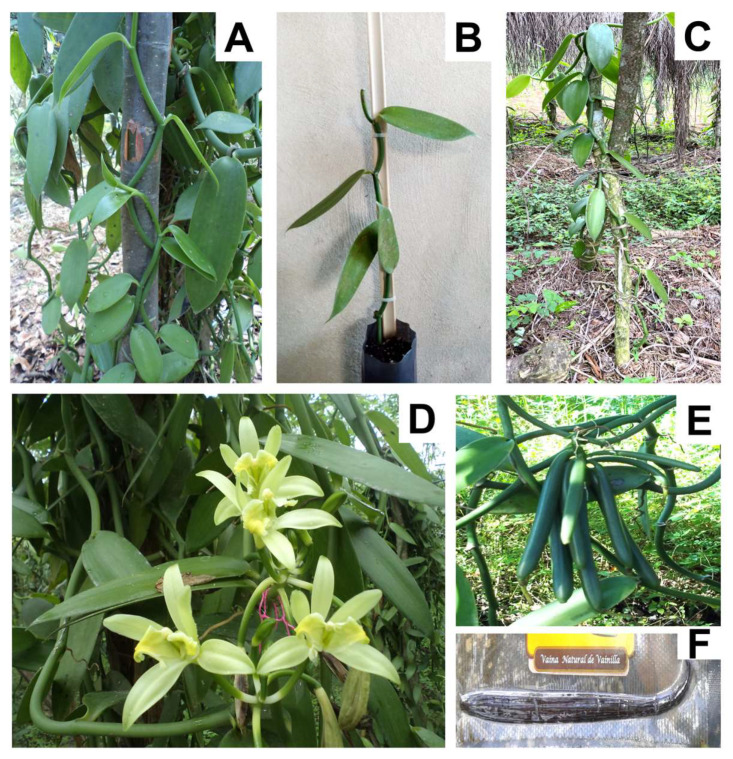
Phenological stages of vanilla plants (*Vanilla planifolia* Andrews). (**A**) Field-grown plants exhibiting multiple stems; (**B**) Traditional vanilla propagation via stem cuttings, (**C**) Commercial plantation establishment using cuttings; (**D**) Vanilla flowering phase; (**E**) Development of dehiscent pods following manual pollination; (**F**) Post-harvest curing and packaging of gourmet vanilla pods for market distribution.

Vanilla (*Vanilla planifolia* Andrews) is a commercially valuable orchid due to the extraction of vanillin from its processed fruits, making it a crop of significant economic importance. However, the current propagation methods are insufficient to meet the demand for propagules for commercial plantations [1]. Conventional propagation through stem cuttings is limited by its inability to produce large quantities of plants, failing to meet the demand for propagator plants [2,3] (Figure 1). Furthermore, sexual propagation via seed germination is hindered by low germination rates, making it an unreliable method [4]. Therefore, developing efficient strategies to increase the propagation of vanilla is crucial. Plant tissue culture (PTC) offers a promising solution, enabling the production of large quantities of plants in a reduced space and short time under controlled, aseptic, and artificial conditions [5].

Plant tissue culture is a technique that enables the mass propagation of diverse plant species [6,7]. This biotechnological approach is applied to plants facing propagation challenges, such as low seed germination rates or inadequate vegetative propagation; economically significant plant species; or phytogenetic resources requiring conservation [8,9]. It is also valuable for producing secondary metabolites of industrial interest and for plant genetic enhancement [10,11]. Furthermore, technological innovations in these tools involve the implementation of temporary immersion systems (TIS); specialized bioreactors designed for plant micropropagation, enabling controlled exposure to liquid media (immersion intervals) via a sterile air supply [12,13]. These TIS offer the advantage of enhancing plant biological yields (biomass production through increased shoot proliferation), while also improving the physiology of the resulting plants by facilitating gas exchange and atmospheric renewal through the supplied air [14,15].

The application of nanomaterials has had a profound impact on various scientific disciplines, including biotechnology. Specifically, nanoparticles (NPs), which range in size from 1 to 100 nanometers, have been explored in plant tissue culture to enhance plant morphological and physiological characteristics [16,17]. Although the exact mechanism of action of NPs is not yet fully understood, their small size allows them to penetrate cells and distribute through vascular tissues, triggering various signaling cascades [18,19]. Notably, metal-based nanomaterials, such as copper nanoparticles (CuNPs), have been shown to be less toxic than their ionic counterparts at equivalent doses [20]. In contrast, silver nanoparticles (AgNPs) exhibit a biostimulant effect in plants, unlike the phytotoxic effect of ionic silver (Ag^+^) [21]. Therefore, further research is necessary to evaluate the effects of nanoparticles from diverse compounds and substances on plant morphology, physiology, and molecular levels, with perspectives of developing plant nanobionics [22,23].

In *V. planifolia*, the application of nanoparticles from various materials has been documented, with silver nanoparticles (AgNPs) being the most commonly used and exhibiting a significant biostimulant effect in temporary immersion systems [24], semi-solid culture systems [25], and evaluations of cytotoxic, genotoxic, and polymorphic effects [26]. However, several nanoparticles remain unexplored in this plant species, particularly those with the potential to enhance biological yields during in vitro propagation. Silicon nanoparticles (SiNPs) have been shown to improve plant tolerance to biotic and abiotic stress by enhancing the expression of antioxidant enzymes, osmoprotectants, proteins, and secondary metabolites such as phenolic compounds [19,27]. Additionally, SiNPs induce non-enzymatic defense responses by increasing the synthesis of ascorbic acid, proline, glutathione, and phytohormones. In in vitro plant propagation, SiNPs have been found to positively influence growth, morphostructural stability, and stress tolerance [28,29,30,31]. Studies have shown that silicon nanoparticles exhibit more pronounced physiological and morphological effects on plants compared to silver nanoparticles [27]. However, the efficacy of these nanoparticles in vanilla cultivation remains unverified. Plant tissue culture has been recognized as a safe and controlled method for studying the effects of nanoparticles on plants, minimizing environmental release and contamination risks [29]. This approach enables precise assessment of nanoparticle impact on plant growth and development, while controlling exposure conditions and variables. By providing a valuable tool for nanoparticle-plant interaction research, plant tissue culture can inform sustainable applications in agriculture and biotechnology. Given the potential benefits of SiNPs, the objective of this study was to evaluate their effect on the micropropagation of *V. planifolia* in RITA^®^ bioreactors.

## 2. Results

### 2.1. Effect of Silicon Nanoparticles (SiNPs) on In Vitro Propagation of Vanilla in RITA^®^ Biorreactors

**Figure 2:**
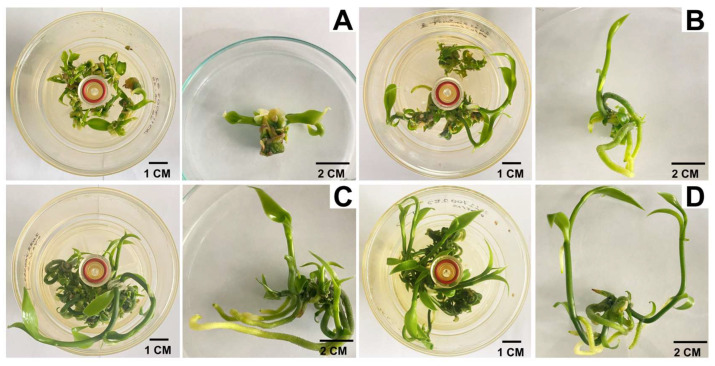
Effect of silicon nanoparticles (SiNPs) after 45 days of in vitro culture of vanilla (*Vanilla planifolia*) in RITA ^®^ bioreactors (Left: inside RITA^®^; Right: outside RITA^®^ (individual explant). (**A**) MS + 0 mg L^−1^ of SiNPs; (**B**) MS + 50 mg L^−1^ of SiNPs; (**C**) MS + 100 mg L^−1^ of SiNPs; (**D**) MS + 150 mg L^−1^ of SiNPs.

After 45 days of culture, significant differences were observed between the evaluated treatments (Table 1). In general, the addition of SiNPs during in vitro propagation of *Vanilla planifolia* in RITA^®^ increased the variables evaluated. An increase in the number of shoots was observed with the increase in SiNP concentration added to the culture medium (Figure 1). The highest number of shoots per explant (5.12) was observed with 150 mg L^−1^ of SiNPs (Figure 2D). By reducing the concentration of SiNPs to 100 mg L^−1^, the formation of 4.66 shoots per explant was observed (Figure 2C), followed by 3.44 shoots per explant in 50 mg L^−1^ (Figure 1B). The lowest number of shoots was observed in the treatment without SiNPs (Figure 2A). This indicates that the addition of SiNPs to the culture medium had a positive effect on the formation of shoots from nodal segments of *V. planifolia* during in vitro propagation in RITA^®^ bioreactors. Regarding shoot length, the addition of SiNPs allowed the production of shoots with greater elongation compared to the treatment without SiNPs. The maximum shoot length of 5.48 cm was achieved with the addition of 150 mg L^−1^ of SiNPs. While reducing the SiNPs concentration to 100 mg L^−1^ resulted in a shoot length of 4.34 cm. Shoot length was significantly reduced to 1.76 cm when 50 mg L^−1^ of SiNPs was added. The shortest shoot length, 1.61 cm, was observed in the treatments with 0 mg L^−1^ of SiNPs. A marked increase in leaf number was observed with the addition of SiNPs compared to the control treatment. The highest leaf formation was observed with the addition of 100 mg L^−1^ of SiNPs, resulting in 10.0 leaves per explant, followed by 9.50 leaves per explant with the addition of 150 mg L^−1^ of SiNPs. In contrast, 50 mg L^−1^ of SiNPs resulted in 8.78 leaves per explant, which was nearly double the number of leaves obtained in the treatment without SiNPs. The lowest number of leaves was observed in the control treatment. One of the most notable effects of SiNPs was the stimulation of root formation and elongation, enabling the simultaneous multiplication and rooting of *V. planifolia* in a single cultivation phase using RITA^®^ bioreactors. SiNPs were notably favored in vitro root formation compared to the control treatment (Figure 2B,C). The maximum root formation, with the highest number of roots per shoot, was achieved with 100 mg L^−1^ of SiNPs in the RITA bioreactors (Figure 2C). In comparison, increasing the SiNP concentration to 150 mg L^−1^ resulted in 5.00 roots per shoot Figure 2D), while decreasing the concentration to 50 mg L^−1^ yielded 3.63 roots per shoot (Figure 2B). No root formation was observed in the control treatment (Figure 2A). Root length was also positively affected by the addition of SiNPs to the culture medium. Root length was maximized at 3.91 cm with the addition of 100 mg L^−1^ of SiNPs. In contrast, root lengths of 3.17 cm and 2.06 cm were observed with 150 mg L^−1^ and 50 mg L^−1^ of SiNPs, respectively. No root formation was observed in 0 mg L^−1^ of SiNPs.

**Figure 3:**
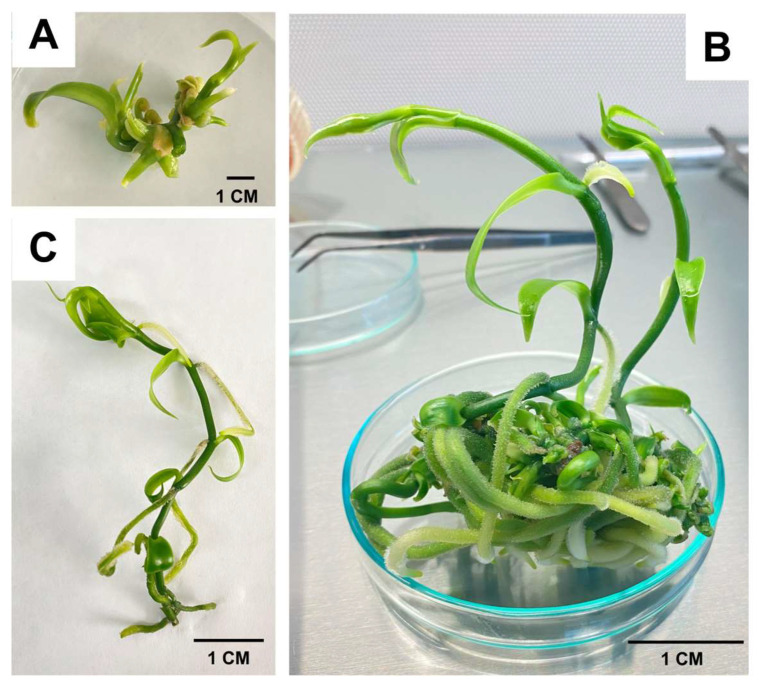
Effect of silicon nanoparticles (SiNPs) on root system formation in vanilla (*Vanilla planifolia*) during in vitro propagation in RITA^®^ bioreactors. (**A**) Shoots with no root system formed in MS + 0 mg L^−1^ of SiNPs; (**B**) Shoots with an adequate root system formed in MS + 150 mg L^−1^ of SiNPs; (**C**) Formation of a large number of roots with great length in MS + 100 mg L^−1^ of SiNPs medium.

The incorporation of SiNPs into the culture medium in RITA^®^ bioreactors significantly enhanced shoot length and root system development (Figure 3B), resulting in a greater number and length of roots (Figure 3C) compared to the control treatment (Figure 3A). This study presents a significant advantage in the large-scale micropropagation of *V. planifolia*, where a high number of roots (5.50–5.63), with considerable length (3.17–3.91 cm), are formed during the shoot proliferation phase. This achievement addresses a major limitation encountered in previous studies, where shoots were abundant but undersized and lacked root development, necessitating an additional phase of elongation and rooting prior to acclimatization (Figure 3).

### 2.2. Acclimatization

**Figure 4:**
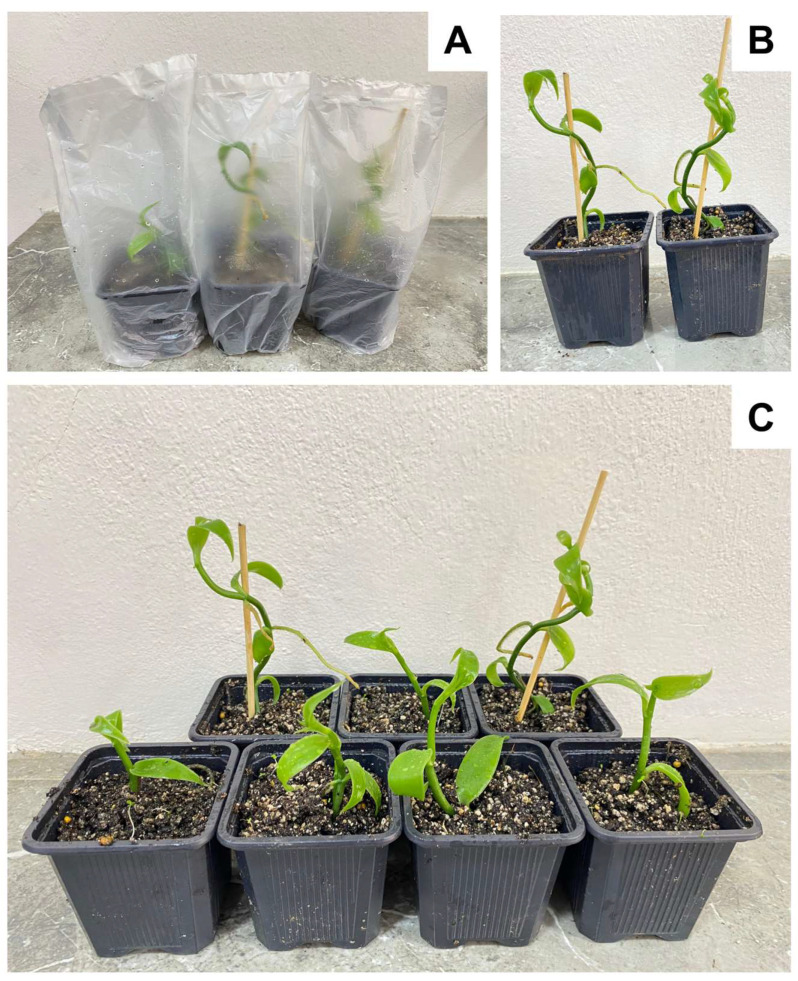
Acclimatization process of *Vanilla planifolia* plants. (**A**) Vanilla plants covered with a plastic bag, maintaining a relative humidity of 95–100%; (**B**) Vanilla plants under ambient relative humidity (40–60%) after 21 days of acclimatization; (**C**) Vanilla plants after 45 days of acclimatization.

The successful formation of a root system in the generated shoots allowed that, after 45 days of acclimatization, a survival rate of 98% was achieved only in plants cultured in RITA^®^ biorreactors (VITROPIC, Saint-Mathieu-de-Tréviers, France) with SiNPs (Figure 4). In contrast, no survival was observed in plants from the treatment without SiNPs due to the absence of root formation and the reduced size of the shoots.

## 3. Discussion

In this study, the use of SiNPs resulted in longer shoots of *V. planifolia* with a greater number and length of roots during micropropagation in temporary immersion systems (RITA^®^ biorreactors). These responses favored high survival rates of the plants obtained during the acclimatization process. The use of nanomaterials in plant tissue culture (PTC) allows for modulation of morphophysiological responses in plants [32,33]. In addition to having positive effects on morphogenesis, callogenesis, metabolism, and seed germination, and improves the synthesis of bioactive compounds [17]. The recent incorporation of nanoparticles (NPs) in plant micropropagation protocols allows for investigation of their effects (positive or negative) in controlled environments and artificial nutrient media [34]. In this context, a thorough evaluation of the type and concentration of NPs used during PTC is required, as a minimal change in quantity can shift from a positive to a negative effect (phytotoxicity) [21,26]. The safe application of nanoparticles (NPs) in plant tissue culture remains uncertain, despite their demonstrated impact on in vitro plant propagation [16]. Research suggests that further investigation is required to elucidate nanoparticle uptake and translocation mechanisms in micropropagated plants, thereby mitigating potential phytotoxic effects and facilitating the development of scalable, sustainable, and precision agricultural systems [35,36]. Silicon has been reported to be effective in in vitro plant propagation, conferring benefits such as modulation of morphogenesis, elicitation of secondary metabolites, and enhanced resistance to abiotic stress [37,38]. However, the application of silicon in nanoparticulate form (SiNPs) remains poorly understood and is a nascent area of research. The present study provides valuable insights into the utilization of silicon nanoparticles (SiNPs) for large-scale plant propagation.

The use of temporary immersion systems (TISs) combined with NPs during in vitro plant propagation allows for greater availability of these substances in the liquid medium and enhances their effect [39,40]. In this study, it was observed that the use of RITA^®^ biorreactors in combination with SiNPs generated the formation of a greater number of roots and increased root length. TISs are culture vessels designed to scale up commercial in vitro plant propagation, specialized for the use of liquid media, separating plant material from the culture medium, and avoiding physiological problems such as hyperhydricity [41,42]. These systems are mediated by a controller, allowing definition of immersion time and frequency [41,43]. The use of RITA bioreactors in the mass micropropagation of *V. planifolia* was addressed by various authors. Ramos-Castellá et al. [44] obtained 14.27 shoots per explant; however, shoot length, number, and length of roots were scarce, resulting in not all explants being able to proceed to acclimatization. Ramírez-Mosqueda and Iglesias-Andreu [45] increased the number of shoots per explant to 18.06 using temporary immersion bioreactors (TIBs); however, they reported a large number of shoots with reduced sizes and the need for a rooting phase to proceed with acclimatization. These particularities are resolved in the present study through the addition of SiNPs at doses of 100 and 150 mg L^−1^. The results obtained in this study suggest that using RITA^®^ biorreactors and SiNPs can generate in vitro rooting and shoot proliferation in a single phase, shortening the production time of vanilla propagules.

The successful use of SiNPs in plant micropropagation was reported in various species. For example, in serpol (*Thymus serpyllum* L.), 200 mg L^−1^ of SiNPs resulted in a higher number of shoots per explant [46]. In this study, the highest shoot number per explant was achieved with 150 mg L^−1^ of SiNPs, suggesting that *V. planifolia* is highly responsive to SiNPs. Notably, this substance has not been previously investigated in this crop, highlighting the potential for novel applications. In lemongrass *Cymbopogon citratus* (DC.) adding exogenous 40 mg L^−1^ of SiNPs in synergy with 4.0 mg L^−1^ 6-Benzyl aminopurine (BAP) and 0.25 mg L^−1^ naphthaleneacetic acid (NAA) increased the number of shoots per explant and eliminated necrosis of the plant material [47]. In *V. planifolia*, the use of SiNPs promoted the generation of a higher number of shoots per explant with increased height and a well-developed root system. Additionally, SiNPs helped mitigate physiological stress in plants grown in liquid media, despite the use of temporary immersion systems [35]. While in the sandalwood tree (*Santalum album* L.), 1.5 mg L^−1^ of SiNPs in combination with plant growth regulators (PGRs) allowed for morphostructural development of the plants, including stem thickening, cortex formation, and vascular cambium differentiation [48]. However, the morphostructural condition of the vanilla plants generated was not analyzed. In the present study, an increase in the number of shoots was observed with increasing SiNPs concentration in the culture medium, as well as root generation and increased root length. Could SiNPs be modulating endogenous auxin levels or improving the plant’s water and nutrient status in vitro, thereby promoting rhizogenesis [21,49]. The synergy between SiNPs and PGRs is thought to be attributed to the functionalization of their silanol groups (Si-OH) with various ligands, enabling conjugation with biologically active molecules and eliciting positive effects on plants [50,51]. Furthermore, mesoporous nanoparticles, characterized by surface pores, are believed to possess enhanced bioactive properties due to their ability to encapsulate and transport functional molecules within cellular structures [52]. The SiNPs employed in this study were functionalized with triethoxylpropylaminosilane, potentially facilitating their fusion with PGRs and subsequent intracellular transport.

Furthermore, previous studies have reported that the use of SiNPs in plant micropropagation can enhance morphostructural development and vigor, facilitating adaptation to ex vitro conditions [31,53]. In this study, high survival rates of the plants obtained were observed, which may be attributed to the morphostructural development of the plants resulting from the addition of SiNPs to the culture medium [54,55]. Future studies could build upon these findings to investigate the potential of SiNPs in mitigating biotic stress in vanilla, thereby expanding our understanding of their applications in this crop. Additionally, the use of temporary immersion systems (TIS) has been shown to favor gas exchange and facilitate acclimatization of the plants obtained [56,57]. Looking ahead, a key challenge in applying SiNPs to in vitro propagated plants is to confer resistance to biotic and abiotic stress factors [19,58]. The strategic incorporation of nanoparticles into in vitro plant propagation programs holds promise for the development of sustainable agriculture, offering a new avenue for improving crop resilience and productivity.

## 4. Materials and Methods

### 4.1. Plant Material

In vitro vanilla (*Vanilla planifolia* Andrews) plants, previously established at the National Center for Genetic Resources-INIFAP from axillary buds, were utilized as the plant material. These plants were derived from the “Mansa” morphotype, native to the Papantla region, Veracruz, Mexico. The plants were initially established in Murashige and Skoog [59] (MS) medium, supplemented with 30 g L^−1^ sucrose and 2.1 mg L^−1^ 6-Benzylaminopurine (BAP, Sigma Chemical Company, MO, USA). 2.5 gL^−1^ Phytagel^TM^ (Sigma-Aldrich, St. Louis, MO, USA) was added as a gelling agent. The pH of the culture medium was adjusted to 5.8 (with 0.1 N sodium hydroxide or 0.1 N hydrochloric acid) and autoclaved at 1.5 kg cm^−2^ and 121 °C for 15 min. The cultures were incubated at 24 ± 2 °C, 16 h light photoperiod and irradiance 50 mmol m^−2^ s^−1^ provided by fluorescent lamps.

### 4.2. Multiplication of Plant

Nodal segments of *Vanilla planifolia* (1 cm in length, containing an axillary bud) were transferred to MS medium supplemented with 30 g L^−1^ of sucrose, 2 mg L^−1^ of BAP (6-Benzylaminopurine). 2.5 g L^−1^ Phytagel^TM^ (Sigma Chemical Company, MO, USA) was used as a gelling agent and pH was adjusted to 5.8 (with 0.1 N sodium hydroxide or 0.1 N hydrochloric acid). Thirty-five milliliters of culture medium were dispensed into glass jars and sterilized in an autoclave at 1.5 kg cm^−2^ and 121 °C for 15 min. The cultures were incubated at 24 ± 2 °C, 16 h light photoperiod and irradiance 50 mmol m^−2^ s^−1^ provided by fluorescent lamps. After 45 days, the multiplication of the plant material was obtained.

### 4.3. Preculture in Liquid Medium

Prior to initiating the experiments in RITA^®^ bioreactor, nodal segments of *V. planifolia* (1 cm in length, containing an axillary bud) were pre-cultured in liquid MS medium without plant growth regulators under partial immersion (~5 mm of the shoot base submerged in liquid medium) for a period of 20 days. This pre-culture allows the explants to improve the adaptation to in liquid medium.

### 4.4. Effect of Silicon Nanoparticles (SiNPs) on In Vitro Propagation of Vanilla in RITA^®^ Biorreactors

Nodal segments (1 cm in length, containing an axillary bud) were transferred to MS medium supplemented with 30 g L^−1^ of sucrose and different concentrations of commercial silicon nanoparticles dispersed in water (concentration of 250 mg mL) (Sigma-Aldrich, St. Louis, MO) (SiNPs: 0, 50, 100, and 150 mg L^−1^). The size of these nanoparticles is <50 nm; they were added directly to the culture medium under constant agitation; subsequently, when poured into the RITA^®^, the same airflow prevented their sedimentation in the liquid medium. The pH of the culture media was adjusted to 5.8 (with 0.1 N sodium hydroxide or 0.1 N hydrochloric acid). RITA^®^ biorreactors (1000 mL, 150 × 130 mm) (VITROPIC, Saint-Mathieu-de-Tréviers, France) with liquid medium (without gelling agent) were used, and 250 mL of culture medium was dispensed into each biorreactor. The culture media were sterilized in an autoclave at 1.5 kg cm^−2^ and 121 °C for 15 min. Ten nodal segments were planted per biorreactor, with five RITA^®^ per treatment. The immersion frequency for the RITA^®^ biorreactors was 2 min every 8 h, according to Ramírez-Mosqueda and Iglesias-Andreu [45]. The cultures were incubated at 24 ± 2 °C, 16 h light photoperiod and irradiance 50 mmol m^−2^ s^−1^ provided by fluorescent lamps. After 45 days of culture, the number of shoots, shoot length, and number of leaves were counted in each of the explants in each treatment. Subsequently, the evaluated variables were expressed as the mean ± standard error of each of the concentrations of SiNPs used.

### 4.5. Acclimatization

Only treatments with SiNPs generated plants 4–5 cm in length with a developed root system (around 200 plants). Subsequently, they were rinsed with tap water to remove residual culture medium. The plants were then planted in a sterile substrate composed of peat moss and perlite (1:3 *v*/*v*) using 2.5-inch pots (7.2 cm × 6.8 cm × 6.8 cm). The plants were transferred to a greenhouse, where they were provided with 90 ± 5% relative humidity, 50% shade, and a temperature of 28 ± 5 °C. To facilitate acclimatization, each pot was covered with a plastic bag to prevent moisture loss. The cover was progressively removed to reduce the relative humidity to environmental levels. Nitrofoska^®^ (1.5 g L^−1^) (N, 25; P, 10; K, 17) (PS, COMPO, Zapopan, Mexico) was applied as foliar fertilizer once a wk, and plantlets were watered three times a week. Irrigation was carried out manually by sprinkling the plants with running water three times a week. This helped maintain a relative humidity level between 60 and 95%. After 45 days of cultivation, the survival rate was evaluated by dividing the total number of plants initially planted by the number of plants that remained alive.

### 4.6. Statistical Analysis

A completely randomized design was used in all experiments. The data obtained were statistically processed using IBM SPSS Statistics software (version 21) (IBM, Armonk, NY, USA). All experiments were conducted twice, with 50 samples per treatment (ten nodal segments were planted per bioreactor, with five RITA^®^). An analysis of variance (one-way ANOVA) followed by a Tukey test (*p* ≤ 0.05) was performed to determine if there were significant differences between treatments. Normality and variance homogeneity were checked by Kolmogorov–Smirnov and Levene tests, respectively. When variables did not show these parameters, they were transformed to natural logarithm (ln).

## 5. Conclusions

Silicon nanoparticles (SiNPs) have been found to positively influence the in vitro rooting of *Vanilla planifolia*. The addition of 150 mg L^−1^ of SiNPs favored the obtainment of shoots with greater length and an optimal root system. These findings contribute significantly to the micropropagation processes of this valuable species, enabling the production of high-quality commercial propagules with a well-developed root system, which will facilitate their acclimatization and subsequent field cultivation. Furthermore, the use of SiNPs in micropropagation protocols may reduce the need for additional rooting phases, thereby streamlining the production process and increasing the efficiency of *V. planifolia* propagation. However, further studies are needed to investigate the various cellular and molecular mechanisms involved in *V. planifolia* responses to SiNPs addition. For instance, measuring endogenous phytohormones, abiotic stress markers (proline, MDA-malondialdehyde, etc.), or the expression of key genes involved in root development and silicon transporters.

## Figures and Tables

**Table 1:** Effect of silicon nanoparticles (SiNPs) on the in vitro propagation of *Vanilla planifolia* in RITA^®^ after 45 days of culture.

SiNPs (mg L^−1^)	No. of Shoots	Shoot Length (cm)	No. of Leaves	No. of Roots	Root Length (cm)
0	2.83 ± 0.40 c	1.61 ± 0.15 c	4.71 ± 0.74 b	0.00 ± 0.00 c	0.00 ± 0.00 d
50	3.44 ± 0.33 bc	1.76 ± 0.32 c	8.78 ± 0.81 a	3.63 ± 0.56 b	2.06 ± 0.31 c
100	4.66 ± 0.23 ab	4.34 ± 0.20 b	10.00 ± 0.57 a	5.63 ± 0.47 a	3.91 ± 0.16 a
150	5.12 ± 0.38 a	5.48 ± 0.22 a	9.50 ± 0.52 a	5.00 ± 0.43 ab	3.17 ± 0.30 b

The values represent the mean ± standard error. Values followed by different letters indicate statistically significant differences according to Tukey’s test (*p* ≤ 0.05).

## Data Availability

The original contributions presented in this study are included in the article. Further inquiries can be directed to the author.
